# Berberine-fluconazole synergy against drug-resistant *Candida albicans*: association with disruption of the Ire1-mediated UPR pathway

**DOI:** 10.3389/fmicb.2026.1965937

**Published:** 2026-10-09

**Authors:** Daifan Yue, Dongming Zheng, Zhen Song, Dongmei Li, Yan Li

**Affiliations:** 1 College of Medical Technology, Chengdu University of Traditional Chinese Medicine, Chengdu, China; 2 Department of Laboratory Medicine, Clinical Laboratory Medicine Research Center, West China Hospital, Sichuan University, Sichuan Clinical Research Center for Laboratory Medicine, Chengdu, China; 3 Department of Nuclear Medicine, Ya’an People’s Hospital, Ya’an, China

**Keywords:** berberine, *Candida albicans*, combination, endoplasmic reticulum, fluconazole

## Abstract

**Objectives:**

*Candida albicans*-associated invasive candidiasis exhibits high mortality. Azole resistance and biofilm-mediated persistent infections greatly weaken clinical antifungal efficacy, necessitating new therapeutic approaches. This study investigated the synergistic antifungal and antibiofilm activities of berberine (BBR) combined with fluconazole (FCZ) against drug-resistant *C. albicans*, and explored potential pathways correlated with this synergistic phenotype.

**Methods:**

Synergistic antifungal effects were verified via fractional inhibitory concentration index and time-kill curve assays. Crystal violet staining and Spider plate culture evaluated biofilm formation and fungal morphology. Transcriptome sequencing was performed to screen potential regulatory pathways and drug targets. Endoplasmic reticulum (ER) homeostasis was assessed by examining ultrastructural changes, misfolded protein accumulation, stress sensitivity and secreted protease activity. *HAC1* splicing and unfolded protein response (UPR)-related gene expression were analyzed to characterize UPR signaling alterations. Autophagy activation was evaluated via ultrastructural observation, vacuole staining, pharmacological inhibition, and detection of autophagy-related gene transcription and Atg1 protein abundance.

**Results:**

BBR combined with FCZ exerted synergistic antifungal effects against drug-resistant *C. albicans* and inhibited biofilm formation. Combined treatment disrupted ER homeostasis and attenuated the Ire1-mediated UPR signaling response in *C. albicans*. It induced autophagy-associated alterations, accompanied by vacuolar structural impairment, up-regulated transcription of autophagy-related genes, and increased Atg1 protein abundance.

**Conclusion:**

The synergistic effect of BBR combined with FCZ is associated with ER stress pathway dysregulation and autophagy activation in *C. albicans*. This study provides a potential therapeutic strategy for azole-resistant candidiasis, while further investigations are required to elucidate the precise underlying molecular mechanisms.

## 1 Introduction

Invasive fungal infections are severe clinical diseases with a steadily rising incidence and a high mortality rate of 45%–75% (Li et al., 2021; Denning, 2024). As an opportunistic pathogen, *Candida albicans* dominates human invasive candidiasis (Bilal et al., 2022; Vannini et al., 2022). In the first list of priority fungal pathogens issued by WHO in 2022, *C. albicans* was classified as one of the four critical priority pathogens, which highlights the urgent demand for new therapeutic strategies (Burki, 2023). Furthermore, *C. albicans* easily adheres to and proliferates on biological surfaces, and the resulting persistent biofilms can markedly elevate the sessile minimum inhibitory concentration (MIC) of antifungals compared with planktonic cells (Vitális et al., 2020). Coupled with the limited therapeutic options for invasive candidiasis, fungal strains have gradually developed resistance to first-line azole drugs, with an incidence of 20%–60% (Parambath et al., 2024). Accordingly, how to effectively restrain the progression of drug resistance has become a key focus and major challenge in the clinical treatment of invasive candidiasis.

The endoplasmic reticulum (ER) is an essential compartment responsible for protein processing, folding and secretion in eukaryotic cells, and is critical for *C. albicans* proliferation and virulence (Walter et al., 2025). When unfolded and misfolded proteins continuously accumulate in the ER lumen, exceeding the intrinsic folding and processing capacity of the ER, this functional imbalance is defined as ER stress (Hetz et al., 2020). To eliminate excessive abnormal proteins, cells activate the unfolded protein response (UPR) to maintain homeostasis (Hetz et al., 2020). In *C. albicans*, only the inositol-requiring enzyme 1 (Ire1)-dependent UPR pathway has been proven to regulate ER stress. This pathway mediates the unconventional splicing of *HAC1* mRNA to generate the functional leucine zipper transcription factor Hac1, which translocates into the nucleus and activates the expression of ER molecular chaperones and protein folding-related enzymes (Travers et al., 2000; Sircaik et al., 2021; Ishiwata-Kimata and Kimata, 2023). Chu et al. (2025) reported that bioinformatic enrichment analysis of the butanol extract of Pulsatilla decoction suggested its potential inhibitory effect on ER function in *C. albicans*, with further RT-qPCR validation confirming the down-regulation of ER-related functional genes. Accumulating evidence shows that ER stress induces autophagy in eukaryotic cells (Senft and Ronai, 2015; Yin et al., 2020). Autophagy helps cells clear aberrant protein aggregates, alleviate ER structure expansion, and eliminate damaged organelles (Bernales et al., 2006; Garcia et al., 2021). ER stress also effectively stimulates the assembly of pre-autophagosomal structures, and expanded ER membrane provides a favorable membrane source for initial autophagosome formation (Yamamoto and Noda, 2020; Gómez-Sánchez et al., 2025). For example, the novel antimicrobial peptide Scyampcin _44–63_ induces ER stress in *C. albicans*. Upon exposure to this peptide, a significant expansion of vacuoles was observed, accompanied by the triggering of autophagic cell death (Zhou et al., 2023). Therefore, modulating key adaptive responses to ER stress and disrupting ER homeostasis may represent a novel and highly promising antifungal strategy.

*Coptis chinensis* Franch., a traditional Chinese herb with cold, bitter properties and a good safety profile, has long been used for diarrhea. Its main active ingredient berberine (BBR) exerts prominent inhibitory effects on a variety of pathogens, including fluconazole (FCZ)-resistant *C. albicans*, methicillin-resistant *Staphylococcus aureus*, and carbapenem-resistant *Klebsiella pneumoniae* (da Silva et al., 2016; Xia et al., 2022; Zhong et al., 2025). Accordingly, it is regarded as a promising candidate agent for developing novel antifungal strategies. Several studies have described multiple antifungal actions exerted by BBR against *C. albicans*, including the inhibition of lanosterol 14α-demethylase (CYP51) activity, disruption of the high-osmolarity glycerol mitogen-activated protein kinase (HOG-MAPK) pathway, and modulation of multidrug-resistance protein 1 (Mdr1p) (Tong et al., 2021; Huang et al., 2022; Zhang et al., 2024). Nonetheless, whether BBR modulates the UPR or induces ER stress in *C. albicans* remains unknown. The precise mechanism of BBR against drug-resistant *C. albicans* warrants further investigation.

In this study, clinically FCZ-resistant *C. albicans* strains were utilized to evaluate whether BBR could improve the therapeutic efficacy of FCZ. Then, transcriptome sequencing was performed to screen candidate targets accompanied by the synergistic antifungal phenotype. The regulatory effects of the combined treatment on the UPR pathway and fungal autophagy responses were investigated. This work aims to characterize the therapeutic potential of BBR combined with FCZ for drug-resistant *C. albicans* infections, and points to the Ire1-mediated UPR pathway as a potential avenue for future development of antifungal strategies against drug-resistant fungal infections.

## 2 Materials and methods

### 2.1 Strains, media, and growth conditions

*Candida albicans* ATCC 10231 was purchased from the Guangdong Microbial Strain Preservation Center. Clinical isolates of *C. albicans* were obtained from the Affiliated Hospital of Chengdu University of Traditional Chinese Medicine and identified as FCZ-resistant strains (CA 1460, CA 2063, CA 2462, CA 2747, CA 42022 and CA 42024). Three isolates (CA1460, CA2063, and CA2462) were randomly selected for subsequent mechanistic experiments. Strains were routinely cultured in yeast extract-peptone-dextrose (YPD) medium containing 1% yeast extract, 2% peptone, and 2% glucose. For solid medium, 2% agar was added. Strains were cultured at 30 °C with shaking at 180 rpm for 18–24 h. For subsequent experiments, *C. albicans* at the logarithmic growth phase were resuspended in RPMI 1640 medium supplemented with 3.5% 3-morpholinopropanesulfonic acid (MOPS, pH 7.0), and the fungal suspension was adjusted to the required concentration. For the biofilm-formation assay in RPMI 1640 medium, the final cell concentration was adjusted to 1 × 10^5^ CFU/mL. To assess filamentation capacity, Spider plates containing 0.2% K₂HPO₄, 1% mannitol, 1% nutrient broth and 2% agar were prepared. A 2 μL aliquot of fungal suspension (1 × 10^6^ CFU/mL) was spotted onto Spider plates. To evaluate the effect of drug intervention on proteolytic activity (Pa), bovine serum albumin (BSA) plates were prepared containing 0.145% yeast nitrogen base without amino acids, 2% glucose, 2% agar, and 0.4% BSA (Li et al., 2014). A 5 μL aliquot of fungal suspension (2 × 10^6^ CFU/mL) was spotted onto BSA plates.

### 2.2 Checkerboard microdilution assay

Experiments were performed according to the Clinical and Laboratory Standards Institute (CLSI) guideline M27-A4 (Wiederhold, 2023). For monotherapy MIC assays, 100 μL serially diluted BBR or FCZ (final concentrations, 256–0.5 μg/mL) was added to each well of a 96-well plate, followed by 100 μL of *C. albicans* suspension to yield a final density of 2 × 10^3^ CFU/mL. For combination MIC assays, serially diluted BBR (final concentrations, 128–0.25 μg/mL) and FCZ (final concentrations, 32–0.25 μg/mL) were dispensed into 96-well plates, and each well was inoculated with *C. albicans* suspension at 2 × 10^3^ CFU/mL. A drug-free positive control and a blank negative control containing only RPMI 1640 medium were set up in parallel. Plates were incubated at 35 °C for 24 h. Afterwards, 20 μL of 2,3-bis-(2-methoxy-4-nitro-5-sulfophenyl)-2H-tetrazolium-5-carboxanilide (XTT) reagent (Keygen, Jiangsu, China) was added to each well, and plates were incubated for another 2 h in the dark. The absorbance at 450 nm (A_450_) was measured with a microplate reader (Molecular Devices, USA). The fractional inhibitory concentration index (FICI) was calculated as follows: FICI = MIC (BBR in combination)/MIC (BBR alone) + MIC (FCZ in combination)/MIC (FCZ alone). FICI results were interpreted based on established criteria (Odds, 2003): FICI ≤ 0.5 indicates synergism; 0.5 < FICI ≤ 4.0 indicates indifference; FICI > 4.0 indicates antagonism.

### 2.3 Time-kill curve assay

Time-kill curve was generated following the method described by Sun et al. (2017). *C. albicans* cells in the logarithmic growth phase were adjusted to a final concentration of 1 × 10^3^ CFU/mL using RPMI 1640 medium, and different concentrations of drugs were added for treatment. The fungal suspension was incubated in a constant-temperature shaker at 30 °C and 180 rpm. At time points of 0 h, 12 h, 24 h, 36 h and 48 h, 200 μL of fungal suspension was collected from each sample. After adding 20 μL of XTT reagent, the mixture was incubated in the dark for 2 h, and A_450_ was subsequently measured. The time-kill curve was plotted with incubation time as the abscissa and the mean absorbance value as the ordinate.

### 2.4 Cytotoxicity assay

The cytotoxicity of BBR against RAW264.7 macrophages was evaluated using the CCK-8 assay (Bioground, Chongqing, China) (Wang et al., 2024). RAW264.7 cells were seeded into 96-well plates at a density of 1 × 10^4^ cells/mL, with 100 μL per well, and incubated overnight at 37 °C in the presence of 5% CO_2_. After incubation, the supernatant was removed and fresh media with BBR were added (concentration ranging from 64 to 1 μg/mL). A drug-free control group and a medium blank group were set up. The plates were incubated for another 24 h. After the supernatant was removed, 100 μL of RPMI 1640 complete medium containing 10% CCK-8 reagent was added to each well. After incubation at 37 °C for 2 h in the dark, the A_450_ of each well was measured.

### 2.5 Crystal violet staining assay

*Candida albicans* suspension was diluted to 2 × 10^6^ CFU/mL with RPMI-1640 medium. A 500 μL aliquot of the fungal suspension was mixed with an equal volume of drug-containing RPMI-1640 medium in a 24-well plate and incubated at 35 °C for 4 h or 24 h. After removal of the medium, each well was stained with 1 mL of 1% (*w*/*v*) crystal violet for 3 min and washed with PBS three times. Samples were destained with 10% (*v*/*v*) acetic acid for 5 min. The absorbance of the eluate at 595 nm was measured using a microplate reader to quantify adherent biomass.

### 2.6 RNA extraction and transcriptome analysis

Samples of *C. albicans* (CA1460) were collected after 24 h of treatment with 1 μg/mL BBR in combination with 1 μg/mL FCZ, along with untreated control samples. Each group consisted of three independent biological replicates. All samples were immediately frozen in liquid nitrogen. Total RNA was extracted using TRIzol reagent (Invitrogen, USA). High-throughput sequencing was performed on an Illumina NovaSeq 6,000 sequencer (Illumina, USA) in PE150 mode. Raw sequencing data were filtered to remove adapter sequences and low-quality reads. The high-quality clean reads were aligned to the *C. albicans* reference genome (SC5314: https://www.ncbi.nlm.nih.gov). Gene expression levels were calculated and presented as Fragments Per Kilobase of exon model per million mapped reads (FPKM). Differentially expressed genes (DEGs) between samples were analyzed using the edgeR software package. DEGs were screened with the thresholds of fold-change ≥ 2 and *q-*value ≤ 0.05. Gene Ontology (GO) functional annotation was performed for the identified DEGs. Significantly enriched GO terms were selected using hypergeometric distribution tests against the whole-genome annotation background. Similarly, Kyoto Encyclopedia of Genes and Genomes (KEGG) pathway enrichment analysis was conducted to identify significantly enriched metabolic pathways using the same statistical method. DEGs were submitted to the STRING online database^1^ for protein–protein interaction (PPI) analysis, and a preliminary PPI network was constructed. The resulting PPI data in TSV format were imported into Cytoscape 3.9 software. Hub genes were identified according to node degree, and the structural features of the PPI network were analyzed.

### 2.7 Molecular docking analysis

The three-dimensional structure of BBR was obtained from the PubChem database.^2^ Target protein information was obtained from the UniProt database,^3^ and the corresponding three-dimensional crystal structures were downloaded from the RCSB PDB database.^4^ Protein structures were imported into Discovery Studio for structural optimization. Both proteins and small molecules were prepared using MGLTools 1.5.6 and saved in PDBQT format for subsequent docking analysis. Molecular docking calculations were performed using AutoDock Vina 2.1.6 to predict the optimal binding conformation and evaluate the binding affinity. Images representing the best poses were prepared using PyMOL software.

### 2.8 Spot assay

Fungal suspensions of *C. albicans* in the logarithmic growth phase were collected and serially diluted with sterile PBS. Dithiothreitol (DTT) was added to YPD medium at final concentrations of 2 mM, 4 mM, and 8 mM. For single or combined drug intervention, the corresponding drugs were supplemented concurrently. A 3.5 μL aliquot of fungal suspension at each dilution gradient was spotted onto the plates. All plates were incubated at 35 °C for 72 h, and colony growth was subsequently observed and recorded.

### 2.9 Misfolded protein level analysis

Misfolded protein levels in *C. albicans* were measured following the method described by Li et al. (2025). Fungal suspension was adjusted to 1 × 10^6^ CFU/mL in RPMI-1640 medium. After the addition of drugs, the suspension was incubated at 30 °C and 180 rpm for 1 h or 24 h. *C. albicans* cells were washed once with PBS and then resuspended. TPE-MI dye (MedChemExpress, USA) was added to a final concentration of 50 μM, followed by incubation at 37 °C for 30 min. After another wash and resuspension in PBS, fluorescence intensity (excitation/emission: 350/470 nm) and A600 values were measured. Mean fluorescence was calculated as TPE-MI fluorescence/A_600_.

### 2.10 *HAC1* mRNA splicing analysis

*Candida albicans* was inoculated in RPMI-1640 medium at an initial density of 1 × 10^6^ CFU/mL and cultured to the logarithmic phase. Drug treatments were performed for 1 h and 24 h. After induction with 4 mM DTT for 1 h, cells were harvested by centrifugation. Total RNA was extracted using TRIzol reagent. cDNA was synthesized using the TransScript First Strand complementary DNA Synthesis SuperMix Kit (Transgen, Beijing, China). Primers were designed as described by Lee et al. (2023). PCR amplification was performed using 2 × Taq PCR MasterMix (Biomed, Beijing, China). The PCR products were separated on a 2% agarose gel at 100 V for 40 min, and images were captured using a gel imaging system. The fluorescence intensity of each band was quantified using Image J, and the ratio of *HAC1_u_* mRNA to (*HAC1_u_* mRNA + *HAC1_i_* mRNA) was calculated (Henry et al., 2024).

### 2.11 4μ8C-mediated Ire1 inhibition assay

*Candida albicans* suspensions were adjusted to 1 × 10^6^ CFU/mL in RPMI-1640 medium and divided into 4μ8C-treated (15 μg/mL) and untreated groups. Cells in both groups were treated with 1 μg/mL BBR combined with 1 μg/mL FCZ or 4 μg/mL BBR combined with 4 μg/mL FCZ. After 1 h of incubation at 30 °C and 180 r/min, fungal cells were collected by centrifugation, resuspended in PBS, and diluted 100-fold. A 5 μL aliquot of the diluted suspension was plated on plates containing 2 mM DTT and incubated at 35 °C for 48 h. Colony growth was observed and recorded.

### 2.12 Fluorescence microscopy analysis

An inverted fluorescence microscope (LEICA, Germany) was used to observe ER and vacuole morphology in *C. albicans*. Cells were adjusted to 1 × 10^6^ CFU/mL in RPMI-1640 medium and treated with drugs for 24 h. After harvesting and washing with PBS, cells were stained with ER-Tracker Blue-White DPX (500 nM, 30 min at 37 °C; Yeasen, Shanghai, China) or C-DCFDA (5 μg/mL, 30 min at 24 °C; Yuanye, Shanghai, China) to visualize ER and vacuole morphology, respectively. Fluorescence images were captured using an inverted fluorescence microscope (400×), and at least five random fields were acquired per group.

### 2.13 Monodansylcadaverine (MDC) staining assay

MDC staining was performed to assess autophagy in *C. albicans* as described by Wu et al. (2023). Cells were adjusted to 1 × 10^6^ CFU/mL in RPMI-1640 medium and treated with drugs for 24 h. After centrifugation, cells were resuspended in PBS and adjusted to 2 × 10^6^ CFU/mL. Staining was conducted using an MDC Autophagy Staining Kit (Beyotime, Shanghai, China). MDC fluorescence intensity was measured using a microplate reader (excitation/emission: 335/512 nm), and fluorescence images were captured under an inverted fluorescence microscope (200×), with at least five random fields per group.

### 2.14 Transmission electron microscopy (TEM)

After 24 h of drug treatment, *C. albicans* cells were collected, washed twice with PBS, and fixed with 3% glutaraldehyde at 4 °C overnight. The cells were then dehydrated through a graded ethanol series and embedded on copper grids. Samples were stained with ethanolic uranyl acetate and lead citrate. Images were acquired using a JEM-1400 FLASH TEM (JEOL, Japan) at 3000× magnification, with at least five random fields captured per group.

### 2.15 Real-time quantitative PCR (RT-qPCR)

*Candida albicans* cells after 24 h of drug treatment were collected by centrifugation (12,000 rpm for 5 min), and total RNA was extracted using TRIzol reagent. cDNA was synthesized using the TransScript First Strand complementary DNA Synthesis SuperMix Kit. qPCR was performed using the TransStart Green qPCR SuperMix Kit (TransGen, Beijing, China). Primers for the target and reference genes were designed using Primer-BLAST (NCBI) and synthesized by Sangon Biotech (Shanghai, China) (primer sequences are listed in Supplementary Table S1). Relative gene expression levels were calculated using the 2^−ΔΔCt^ method.

### 2.16 Western blotting

Total protein was extracted from the cell pellets using a Yeast Protein Extraction Kit (Keygen, Jiangsu, China) combined with an ultrasonic homogenizer (Scientz, Ningbo, China). Protein concentration was determined using the BCA Protein Assay Kit (Beyotime, Shanghai, China). Prepared protein samples were separated by molecular weight using SDS-PAGE on a 10% PAGE gel (Vazyme, Nanjing, China) and transferred onto a PVDF membrane. After blocking, membrane was incubated with the appropriate antibody (Supplementary Table S2). Chemiluminescence reactions were performed using an ultrasensitive ECL Kit (4A Biotech, Beijing, China), and protein bands were imaged using an electronic pressure imaging system (e-Blot, Shanghai, China). Band grayscale intensities were quantified using Image J software.

### 2.17 Statistical analysis

Statistical analyses were performed using SPSS 27.0. Differences between two groups were assessed by Student’s two-tailed *t*-test. For multiple-group comparisons, one-way analysis of variance (ANOVA) was used, followed by the Bonferroni post-hoc test for pairwise comparisons when the overall ANOVA was significant. All experiments were performed with three independent biological replicates, with three technical replicate wells per replicate. Quantitative data were expressed as mean ± standard deviation. A *p* value < 0.05 was considered statistically significant.

## 3 Results

### 3.1 Synergistic antifungal and antibiofilm activities under BBR-FCZ combination treatment in *Candida albicans*

Under monotherapy, the MIC of FCZ against six clinical isolates was >256 μg/mL (exceeding the maximum test concentration on checkerboard plates), while the MIC of BBR was 64 μg/mL (Table 1 and Figure 1A). The MIC values of FCZ and BBR for the reference strain *C. albicans* ATCC 10231 were 1 μg/mL and 32 μg/mL, respectively. Combination treatment lowered the MICs of BBR and FCZ to 0.5–1 μg/mL. The MIC of FCZ decreased more than 256-fold. Accordingly, the obtained FICI values represented conservative upper-bound estimates, and all upper-bound FICI values were less than 0.020 (FICI ≤ 0.5 is defined as synergy). These data therefore supported the conclusion of antifungal synergy between the two agents. The synergistic concentration was the combination of 1 μg/mL BBR and 1 μg/mL FCZ. Considering that high-dose strategies are commonly used to treat drug-resistant and biofilm-associated infections, a high-concentration combination group (4 μg/mL BBR + 4 μg/mL FCZ) was additionally included. The time-kill curve showed that both combination groups maintained a consistently low slope within 0–24 h, indicating sustained synergistic antifungal effects (Figure 1B). RAW264.7 macrophage viability remained above 80% at BBR concentrations of 1–16 μg/mL (Figure 1C). The combination treatment also exhibited favorable inhibitory effects in biofilm-related experiments. At the 4 h adhesion stage and 24 h biofilm formation stage, crystal violet eluate absorbance in the two combination groups was markedly lower than that in the blank control and matching monotherapy groups (Figure 1D). On Spider plates, well-extended hyphae were observed at the colony edges of the blank control and BBR monotherapy groups (Figure 1E). No obvious hyphae were found around colonies in the combination groups. After 48 h incubation in RPMI-1640 liquid medium, hyphae in the blank control group continued to elongate and interweave into a network, suggesting biofilm formation (Figure 1F). In contrast, the combination groups showed only a slight increase in fungal cell density, with no further hyphal induction.

**Table 1:** Inhibitory activity of BBR combined with FCZ against clinical isolates of *C. albicans.*

Strain	MIC (μg/mL) alone		MIC (μg/mL) in combination		FICI	Interpretation
	FCZ	BBR	FCZ	BBR		
CA 1460	>256	64	1	1	<0.020	Synergy
CA 2063	>256	64	1	1	<0.020	Synergy
CA 2462	>256	64	1	1	<0.020	Synergy
CA 2747	>256	64	0.5	1	<0.018	Synergy
CA 42022	>256	64	1	1	<0.020	Synergy
CA 42024	>256	64	1	0.5	<0.012	Synergy

*C. albicans* ATCC 10231 was used as a reference strain, and the MIC values for FCZ and BBR were 1 μg/mL and 32 μg/mL, respectively.

**Figure 1:**
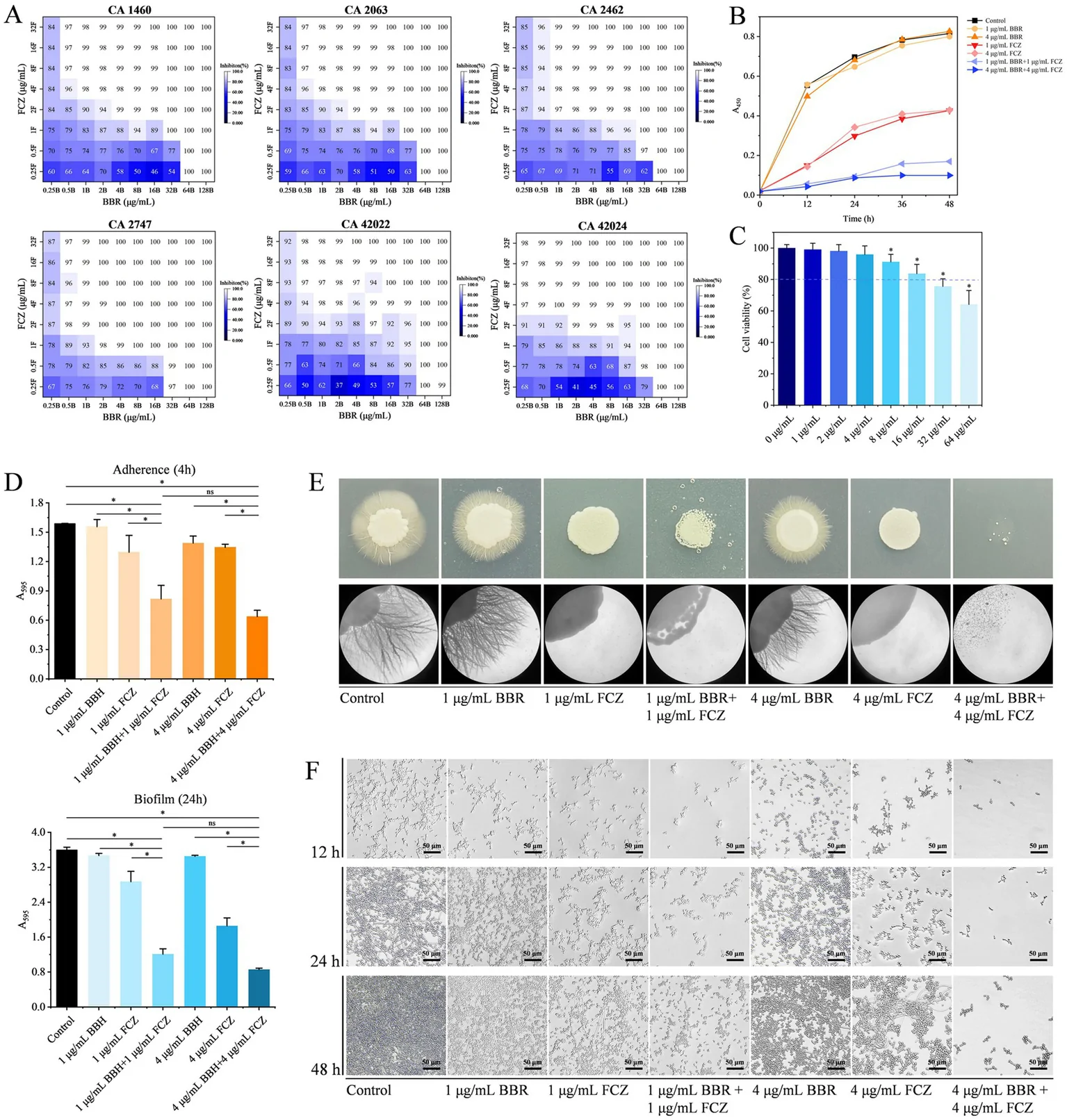
Synergistic inhibition of growth and biofilm formation in *C. albicans* by BBR combined with FCZ **(A)** Checkerboard microdilution heatmap of drug combination against six clinically isolated strains (CA 1460, CA 2063, CA 2462, CA 2747, CA 42022, and CA 42024). The values in each grid represent the inhibition rate (%), and dark blue indicates a lower inhibition rate. **(B)** Dynamic synergistic effect of the combined treatment against *C. albicans* within 0–48 h assessed by the XTT reduction assay. **(C)** Cell viability of RAW264.7 macrophages after 24 h of treatment with 1–64 μg/mL BBR determined by the CCK-8 assay, for the cytotoxicity evaluation of BBR. **(D)** Effects of drug intervention on the adhesion capacity (4 h) and biofilm formation ability (24 h) of *C. albicans*. *C. albicans* cells were stained with 1% crystal violet solution and the A_595_ of the supernatant was measured. **(E)** Colony and hyphal growth of *C. albicans* after 5 days of culture on Spider medium (0.2% K₂HPO₄, 1% mannitol, 1% nutrient broth and 2% agar powder). **(F)** Hyphal derivation and biofilm formation of *C. albicans* after 12 h, 24 h, and 48 h of culture in RPMI-1640 liquid medium.

### 3.2 Transcriptomic analysis of *Candida albicans* under BBR-FCZ combination treatment

Transcriptome sequencing identified a total of 2,128 significant DEGs (1,637 upregulated, 491 downregulated). GO enrichment analysis (Figure 2A and Supplementary Table S3) revealed enrichment of the terms “perinuclear endoplasmic reticulum” and “protein metabolic process”. The core function of the ER, “lipid biosynthetic process”, and numerous terms related to oligopeptide, dipeptide, and tripeptide transport are also significantly enriched. KEGG enrichment analysis (Figure 2B and Supplementary Table S4) showed significant enrichment of metabolic pathways such as “steroid biosynthesis”, “sphingolipid metabolism”, “glycosyltransferases”, and “GPI-anchored proteins”. An ER-related DEGs set was selected to construct a PPI network (Figure 2C). Among all nodes, the ER chaperone protein Kar2 exhibited the highest degree, followed by the protein translocation channel protein Sec61. Meanwhile, inositol-requiring enzyme Ire1 and ER oxidoreductase Ero1 showed interaction edges with stronger connection strength to Kar2 in the PPI network. Then, Kar2, Sec61, Ero1, and Ire1 were selected for in silico molecular docking simulation. Molecular docking simulations (Figure 2D) predicted potential binding between BBR and Kar2, Sec61, Ero1, and Ire1, with estimated binding energies of −8.7 kcal/mol, −7.8 kcal/mol, −6.3 kcal/mol, and −8.9 kcal/mol, respectively.

**Figure 2:**
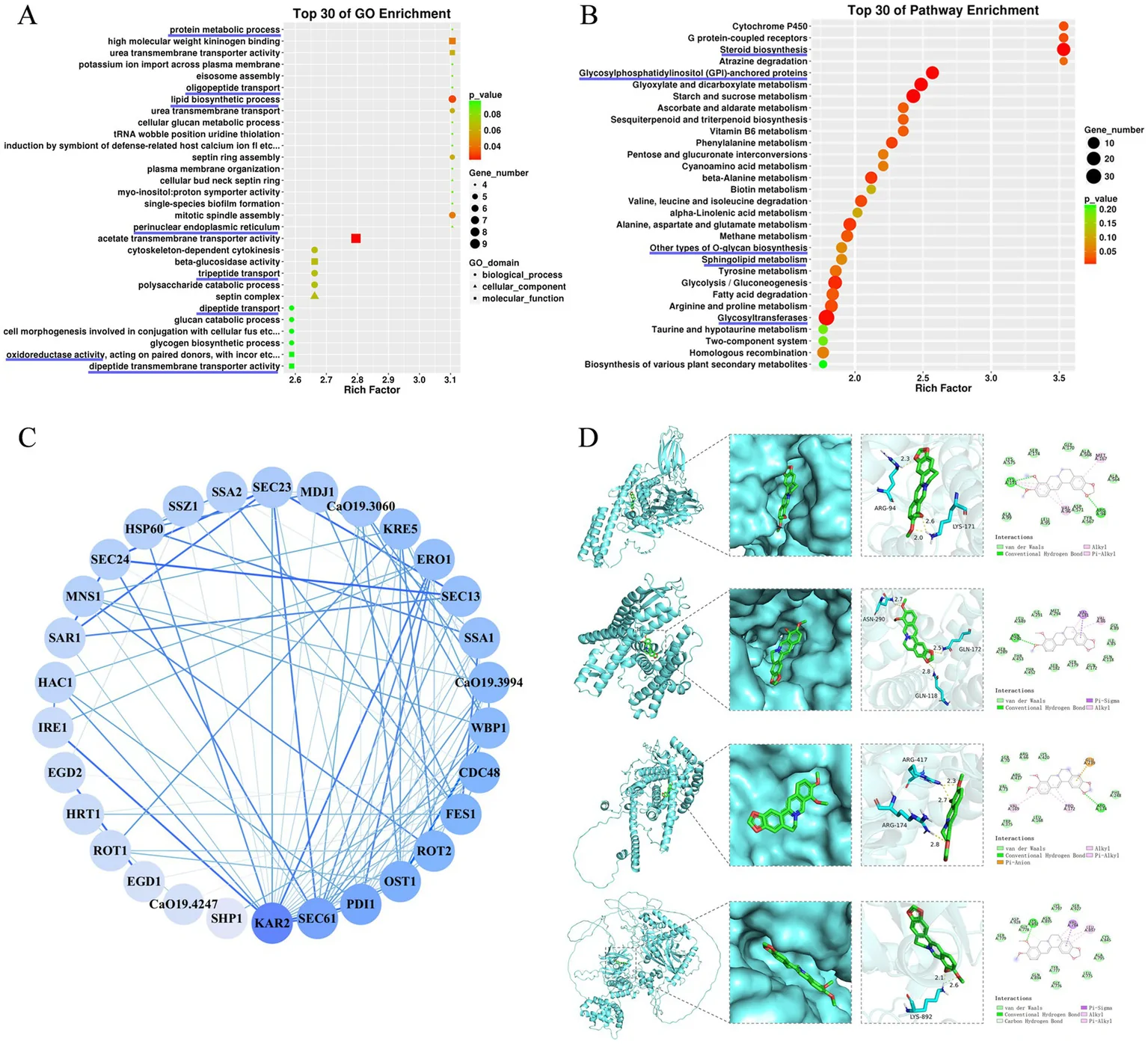
Transcriptomic analysis of *C. albicans* following combined treatment with BBR and FCZ **(A)** Top 30 GO functional terms with the highest enrichment degrees among the DEGs. **(B)** Top 30 KEGG functional terms with the highest enrichment degrees among the DEGs. **(C)** PPI network of ER-related DEGs constructed using Cytoscape software. Nodes represent proteins, and edges indicate protein–protein interactions. Nodes closer to the network center showing darker colors. **(D)** Optimal binding modes between BBR and Kar2, Sec61, Ero1, and Ire1. The molecular structure of BBR is shown in green. Each subgraph includes the cavity structure of molecular docking, a partial enlarged view of the binding sites, and a two-dimensional schematic diagram.

### 3.3 Disrupted ER homeostasis in *Candida albicans* under BBR-FCZ combination treatment

Based on transcriptomic findings, strain susceptibility to ER stress was assessed (Figure 3A). Combination-treated strains displayed higher sensitivity to DTT, and growth inhibition of drug-resistant *C. albicans* became more pronounced with increasing DTT concentrations. ER morphology was visualized via DPX staining (Figure 3B). Quantification revealed that the synergistic combination group exhibited 2.03-, 1.86-, and 1.47-fold higher mean DPX fluorescence intensity than the blank control, 1 μg/mL BBR, and 1 μg/mL FCZ groups, respectively. A similar trend was observed for the high-dose combination group (Figure 3C). Abnormal accumulation of misfolded proteins is one of the typical features of ER stress, and misfolded protein levels were evaluated using TPE-MI dye (Figure 3D). At 1 h, misfolded protein levels in both the synergistic and high-dose combination groups were significantly higher than those in the blank control and corresponding-concentration BBR groups. At 24 h, both combination groups exhibited significantly elevated misfolded protein levels relative to their respective control and monotherapy groups. Misfolded protein accumulation was also time-dependent (Figure 3E). Since secreted protease function depends on the ER-mediated protein transport system, protease activity was analyzed on BSA plates. The combination groups exhibited smaller hydrolysis zones than the blank control and monotherapy groups (Figure 3F). The Pa values of the synergistic combination group and high-dose combination group were 0.70 and 0.94, respectively. Both values were significantly higher than those of the blank control and corresponding monotherapy groups, indicating reduced secreted protease activity (Figure 3G).

**Figure 3:**
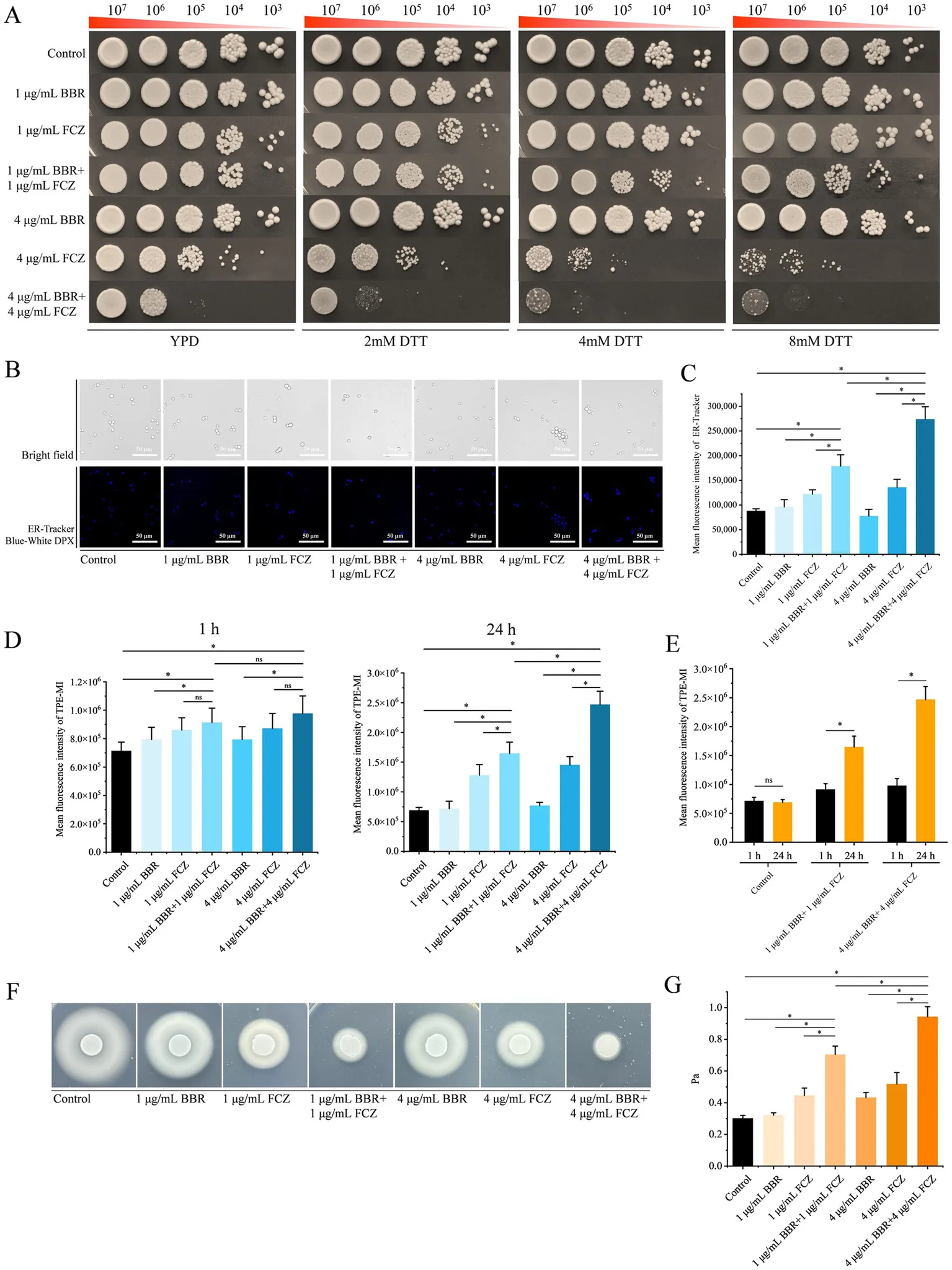
Effects of BBR combined with FCZ on ER homeostasis in *C. albicans* **(A)** Colony growth phenotype of *C. albicans* cultured on YPD solid medium containing DTT at concentrations of 2 mM, 4 mM, and 8 mM for 72 h. **(B)** Fluorescence microscopy images of *C. albicans* stained with ER-Tracker Blue-White DPX. At least five microscopic fields were captured at 400 × magnification; scale bar = 50 μm. **(C)** Mean fluorescence intensity of *C. albicans* stained with ER-Tracker Blue-White DPX (excitation/ emission wavelength: 374/560 nm). **(D)** Intracellular levels of misfolded proteins in *C. albicans* evaluated by TPE-MI staining after drug intervention for 1 h and 24 h (excitation/ emission wavelength: 350/470 nm). **(E)** Intracellular levels of misfolded proteins in *C. albicans* assessed by TPE-MI staining after combined drug intervention for 1 h and 24 h (excitation/emission wavelength: 350/470 nm). **(F)** Growth of *C. albicans* on BSA plates after 72 h incubation. **(G)** Pa values of *C. albicans* following drug treatment. Pa = b/a, where *a* is the sum of the colony diameter and hydrolysis zone diameter, and b is the diameter of the colony alone. A higher Pa value indicates lower enzymatic activity.

### 3.4 Changes in Ire1-mediated UPR pathway activity in *Candida albicans* under BBR-FCZ combination treatment

The UPR is the major pathway for alleviating ER stress in *C. albicans*, and *HAC1* mRNA splicing is a key indicator of UPR activation. Given the increased misfolded protein levels at 1 h and 24 h after combination treatment, *HAC1* mRNA splicing was further analyzed. As shown in Figures 4A,B, *HAC1* mRNA splicing efficiency was significantly lower in both the synergistic combination group and high-dose combination group than in control and corresponding monotherapy groups at 1 h, and this trend persisted at 24 h. Meanwhile, *HAC1* mRNA splicing activity in BBR and FCZ monotherapy groups decreased significantly over time, which may be associated with compensatory regulation induced by long-term drug exposure. The Ire1 inhibitor 4μ8C was used to evaluate the survival of drug-resistant *C. albicans* under stress and thereby reflect the functional status of the UPR pathway. As shown in Figures 4C,D, 4μ8C intervention reduced colony counts by 31.31% in the synergistic combination group and by 79.57% in the high-dose combination group relative to pre-intervention values. *IRE1* expression in the synergistic combination group was significantly down-regulated to 0.79-, 0.84- and 0.66-fold relative to the blank control, 1 μg/mL BBR and 1 μg/mL FCZ groups, respectively (Figure 4E). In the high-dose combination group, *IRE1* expression decreased to 0.48-, 0.52- and 0.39-fold compared with the blank control, 4 μg/mL BBR and 4 μg/mL FCZ groups, respectively. Furthermore, UPR-related genes including *KAR2*, *SEC61*, and *ERO1* exhibited significant down-regulation under high-dose combination treatment.

**Figure 4:**
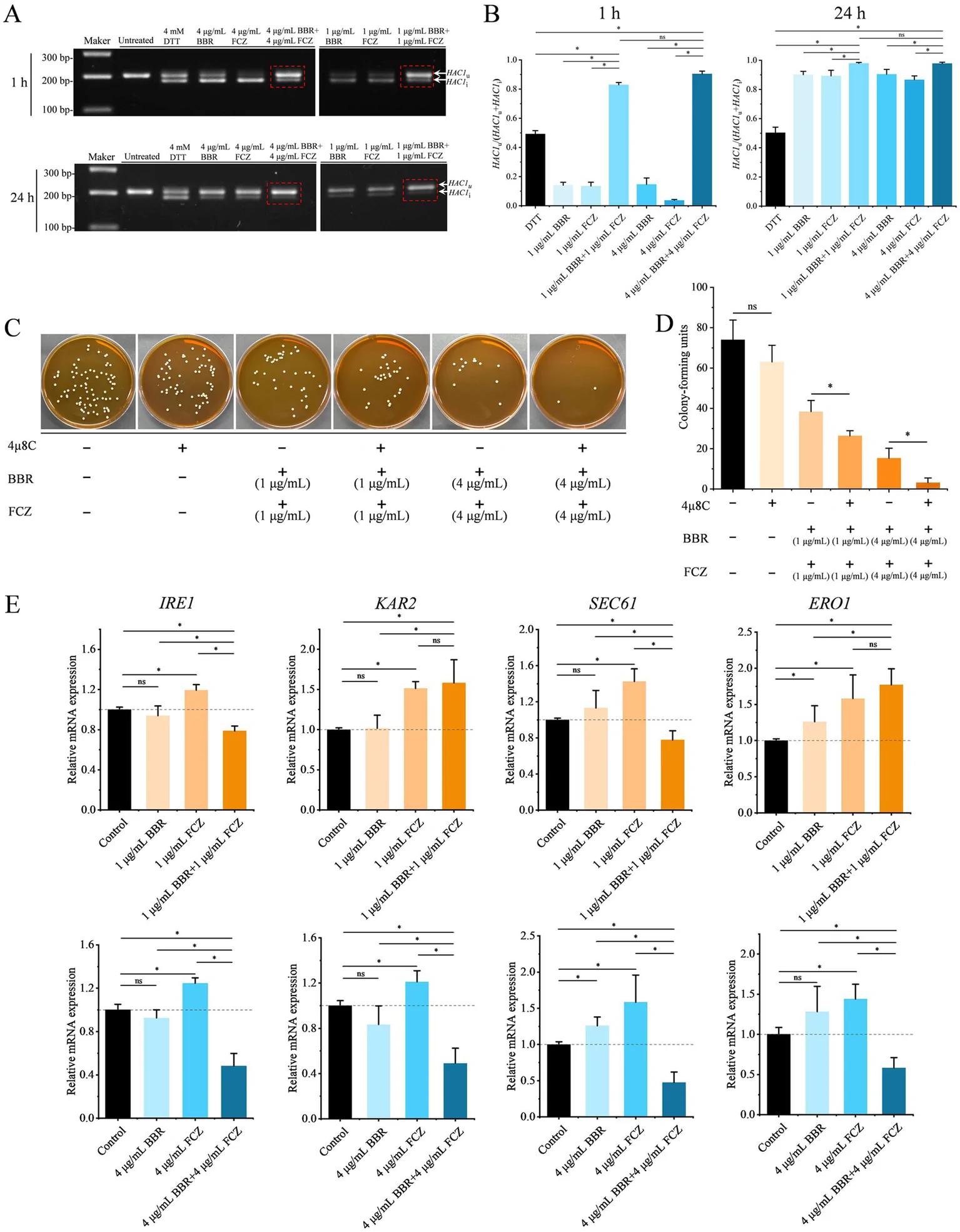
Effects of BBR combined with FCZ on the Ire1-dependent UPR pathway in *C. albicans* **(A)** 2% agarose gel electrophoresis of PCR products after drug treatment for 1 h and 24 h (100 V, 40 min). “*u*” indicates the unspliced form, and “*i*” indicates the spliced form. **(B)** Agarose gel electrophoresis images were analyzed using Image J software to calculate the ratio of *HAC1*_u_ mRNA/ (*HAC1*_u_ mRNA + *HAC1*_i_ mRNA). **(C)** Colony survival phenotypes of *C. albicans* under 4μ8C intervention. **(D)** Quantitative analysis of colony survival of *C. albicans* under 4μ8C intervention. **(E)** Total RNA was extracted after 24 h of drug intervention, and the mRNA expression levels of UPR-related genes (*IRE1*, *KAR2*, *SEC61*, and *ERO1*) in drug-resistant *C. albicans* were determined by RT-qPCR.

### 3.5 Autophagy-associated responses in *Candida albicans* under BBR-FCZ combination treatment

When cells fail to restore homeostasis via the UPR, autophagic degradation process is initiated (Song et al., 2017; Cao et al., 2021). MDC staining (Figures 5A,B) revealed significantly enhanced fluorescence intensity in the combination groups compared with the control and corresponding-concentration BBR groups. Ultrastructural observation by TEM further provided morphological evidence of autophagy (Figure 5C). Unlike the intact structure in the blank control group, combination treatment led to indistinct cell walls and plasma membranes, markedly enlarged cytoplasmic vacuoles, and observable intravacuolar autophagic bodies. Partial vacuole disruption and abundant small membrane vesicles were also detected in the high-dose combination group (Figure 5D). C-DCFDA staining was performed to label the vacuolar structure of *C. albicans*. Both the synergistic and high-dose combination groups exhibited diffuse fluorescence and impaired vacuolar morphology, with a higher proportion of C-DCFDA-positive cells than control and their corresponding monotherapy groups (Figures 5E,F). To explore the association between combination treatment-related autophagic responses and fungal survival, the autophagy inhibitor 3-methyladenine (3-MA) and activator rapamycin (RAPA) were applied. Treatment with 3-MA significantly increased cell viability by 11.30% in the high-dose combination group compared with the corresponding group without 3-MA (Figure 5G). In contrast, RAPA supplementation further decreased cell viability in both combination groups relative to their respective counterparts without RAPA (Figure 5H).

**Figure 5:**
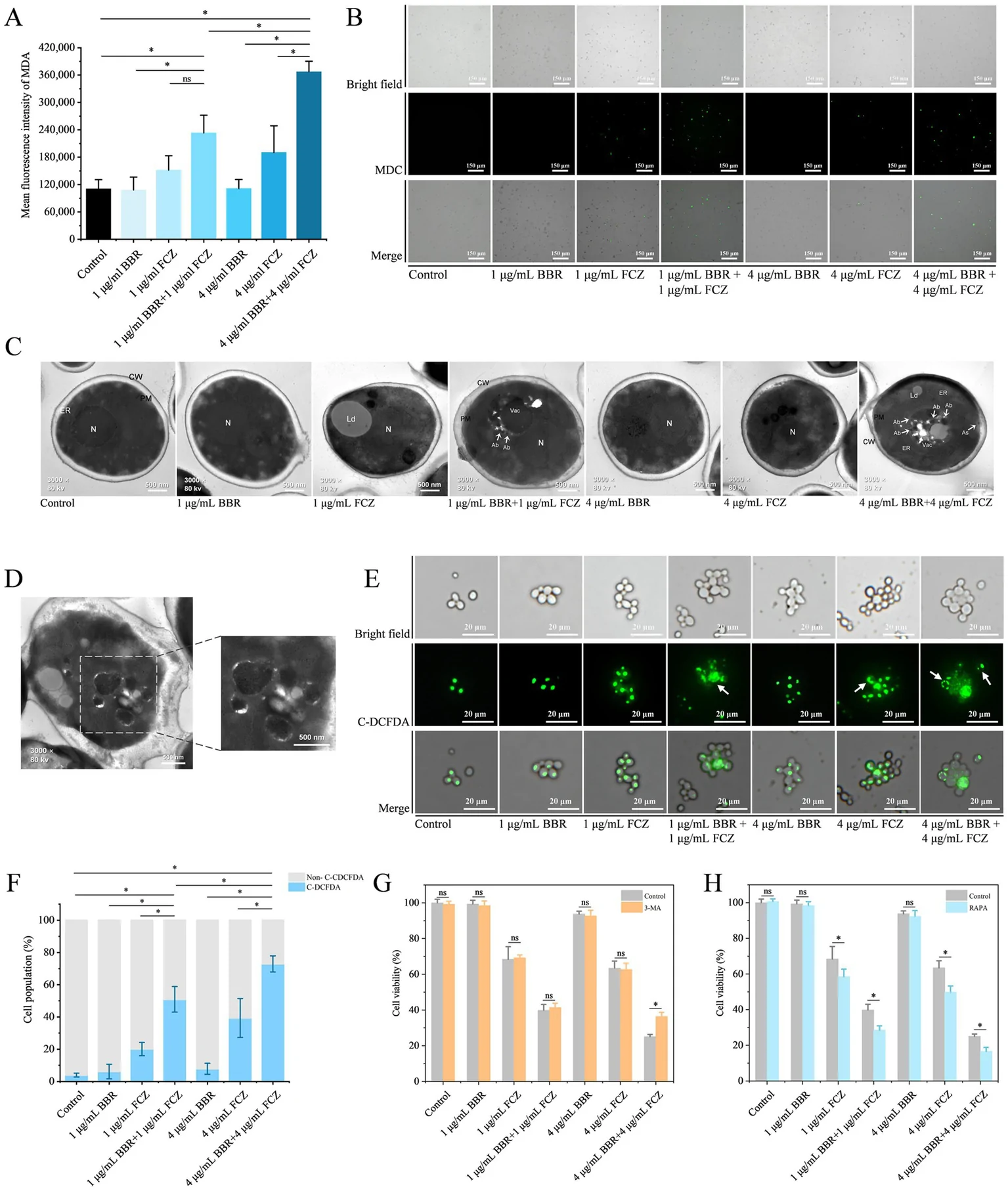
Effects of BBR combined with FCZ on autophagy in *C. albicans* **(A)** Mean fluorescence intensity of MDC-stained *C. albicans* after 24 h of drug treatment (excitation/ emission wavelength: 335/512 nm). **(B)** Fluorescence microscopic images of MDC-stained *C. albicans*. At least five microscopic fields were observed at 200× magnification; scale bar = 150 μm. **(C)** TEM images of *C. albicans* after 24 h of drug intervention (3000×). N, nucleus; PM, plasma membrane; CW, cell wall; ER, endoplasmic reticulum; Ld, lipid droplet; Vac, vacuole; Ab, autophagic body; As: autophagosome; scale bar = 500 nm. **(D)** TEM images showing vacuole fragmentation induced by high-dose combination treatment. **(E)** Fluorescence microscopic images of C-DCFDA-stained *C. albicans* after 24 h of drug treatment. White arrows indicate the diffusely distributed fluorescent signals. At least five microscopic fields were observed at 400 × magnification; scale bar = 20 μm. **(F)** Percentage of C-DCFDA-positive cells. **(G)** Changes in cell viability of *C. albicans* affected by the autophagy inhibitor 3-MA assessed via the XTT reduction assay. **(H)** Changes in cell viability of *C. albicans* affected by the autophagy activator RAPA assessed via the XTT reduction assay.

### 3.6 Expression changes of autophagy-related genes and Atg1 protein in *Candida albicans* under BBR-FCZ combination treatment

The effects of combination treatment on the expression of autophagy-related genes are shown in Figure 6A. For *ATG1*, expression was upregulated to 1.30-fold in the synergistic combination group relative to the blank control group. In the high-dose combination group, *ATG1* expression was upregulated to 1.78-, 1.41-, and 1.55-fold relative to the blank control, 4 μg/mL BBR, and 4 μg/mL FCZ groups, respectively. No significant alteration in *ATG2* expression was observed. For *ATG8* and *ATG12*, expression levels were upregulated in both the synergistic and high-dose combination groups compared to the control and corresponding-concentration BBR groups. The Atg1 kinase complex, an upstream component of autophagy, exhibits high activity during ER stress-induced autophagy (Yorimitsu et al., 2006). Atg1 protein abundances were determined by Western blot (Figures 6B,C). In the synergistic combination group, Atg1 levels were 1.35- and 1.19-fold significantly higher than those in the blank control and 1 μg/mL BBR groups, respectively. In the high-dose combination group, Atg1 levels were 2.20-, 1.70-, and 1.22-fold significantly higher than those of the blank control, 4 μg/mL BBR, and 4 μg/mL FCZ groups, respectively.

**Figure 6:**
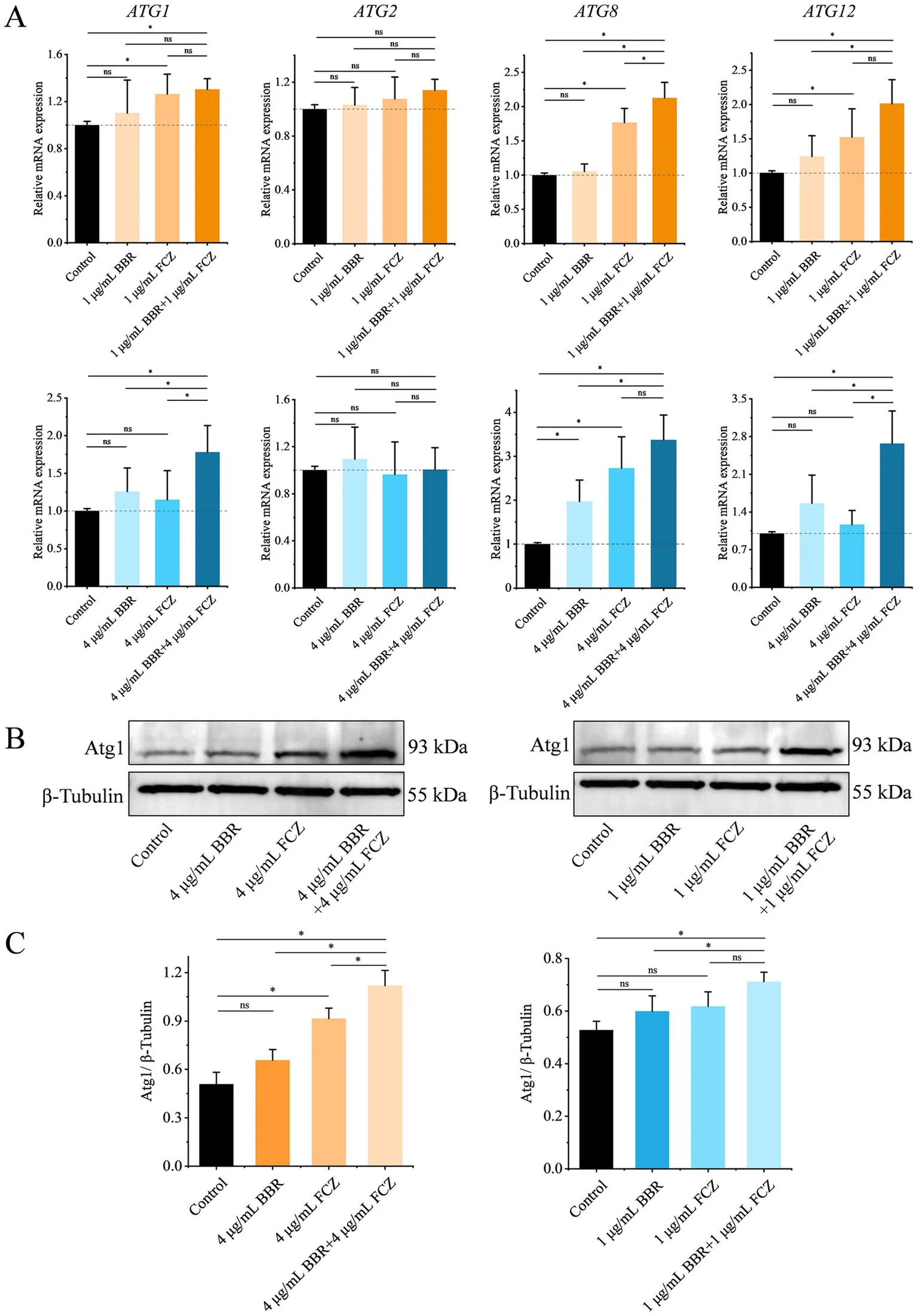
Effects of BBR combined with FCZ on the expression of autophagy-related genes and Atg1 protein in *C. albicans* **(A)** Total RNA was extracted after 24 h of drug treatment, and the mRNA expression levels of autophagy-related genes (*ATG1*, *ATG2*, *ATG8* and *ATG12*) in drug-resistant *C. albicans* were determined by RT-qPCR. **(B)** Total protein was extracted after 24 h of drug treatment, and the protein bands of Atg1 were detected by Western blot. **(C)** The gray values of Atg1 protein bands were analyzed using Image J software and normalized to β-Tubulin as an internal control. The relative expression level of Atg1 was calculated as the ratio of Atg1 gray value to β-Tubulin gray value.

## 4 Discussion

In recent years, researchers have been committed to exploring novel drug resistance mechanisms and developing alternative therapeutic strategies to combat drug-resistant fungal infections. Compared with monotherapy, combination therapy offers multiple advantages, including reduced drug dosages, decreased host toxicity, and delayed development of drug resistance, providing promising clues for identifying novel drug action patterns. Therefore, the present study sought to explore the synergistic antifungal activity of BBR combined with FCZ against drug-resistant *C. albicans* and its potential mechanisms. Given the FCZ monotherapy MIC exceeding 256 μg/mL, all calculated FICI values were below 0.020, representing a conservative upper-bound estimate. Even so, the results still support the synergistic antifungal effect of BBR combined with FCZ against drug-resistant *C. albicans*. BBR at 1–4 μg/mL did not significantly reduce RAW264.7 macrophage viability, providing preliminary safety evidence for the clinical application of BBR. Tong et al. (2021) previously reported that the cytotoxic concentrations of BBR against human HepG2 hepatocytes, NIH/3 T3 fibroblasts, and 293 T renal cells were higher than 90 μg/mL, 100 μg/mL, and 80 μg/mL, respectively. Kou et al. (2017) reported that BBR exerted no obvious inhibitory effect on the viability of THP-1 cells at concentrations up to 70 μg/mL. These findings suggest that BBR exerts obvious inhibitory effects on mammalian cell growth only at relatively high doses. Furthermore, the combination treatment effectively inhibited the early adhesion and mature biofilm formation of *C. albicans*, further reducing the risk of clinical drug resistance. These *in vitro* findings suggest that BBR may serve as a promising adjuvant agent to improve the antifungal efficacy of FCZ. However, additional *in vivo* studies are needed to validate the therapeutic efficacy and safety of this combination regimen. In follow-up work, we plan to establish an invasive candidiasis model and assess the *in vivo* antifungal activity of this regimen by measuring fungal burden, survival curves, and histopathological changes.

Transcriptome sequencing and bioinformatic enrichment analyses were performed to screen targets and signaling pathways potentially associated with the synergistic effect. DEGs-enrichment analysis yielded findings generally consistent with previous reports. In *S. cerevisiae*, downstream regulatory genes of the UPR include ER-resident molecular chaperones, as well as genes associated with translocation, protein glycosylation, GPI anchoring, cell wall biosynthesis, and sphingolipid biosynthesis (Travers et al., 2000). Sircaik et al. (2021) compared transcriptomic profiles between wild-type and *IRE1* diminished expression mutant strains of *C. albicans*. The *IRE1* mutant showed downregulated genes related to metabolic processes, protein synthesis, translation, and ribosome biogenesis. Meanwhile, processes dependent on functional protein export were also perturbed, as evidenced by the down-regulation of genes involved in cell-surface remodeling, biofilm formation, and hyphal growth. Furthermore, genes related to oxidative stress, pH alteration, and nutritional stress were significantly up-regulated (Sircaik et al., 2021). Based on these exploratory transcriptomic data, PPI network construction and in silico molecular-docking simulations were further performed. Among the candidate proteins, Ire1 exhibited the highest predicted binding affinity for BBR. It should be noted that these computational outcomes can only provide preliminary clues for subsequent mechanistic studies, and additional genetic validation is still required in future investigations.

DTT specifically disrupts protein disulfide bond formation, and *IRE1*-deficient yeast cells are sensitive to sustained DTT-induced ER stress (Halbleib et al., 2017). Here, DTT was used as an ER stress inducer, and the drug combination was observed to increase the susceptibility of strains to ER stress. Subsequently, ER stress status was assessed by examining ER morphology, misfolded protein levels and secreted protease activity. ER-Tracker staining showed that combination treatment induced ER morphological expansion. As reported previously, yeast ER volume can expand at least 5-fold under stress, providing extra space for protein folding to maintain homeostasis and alleviate aberrant protein aggregation (Bernales et al., 2006). In this work, misfolded protein levels increased with longer treatment time. Excessive accumulation of misfolded proteins can arrest cell growth in *S. cerevisiae* (Haynes et al., 2004). Disruption of the Erg11-Ncp1 interaction also promotes misfolded protein accumulation, thereby increasing azole susceptibility in *C. albicans* (Li et al., 2025). These experimental observations point toward perturbation of ER homeostasis upon combination treatment.

The functional state of ER stress adaptive pathways was further explored at the molecular level. ER stress and UPR activation are often confused but represent distinct biological processes. ER stress represents disrupted ER homeostasis, whereas the UPR is an adaptive program activated to restore cellular equilibrium (Acosta-Alvear et al., 2025). In *C. albicans*, the UPR is mediated by Ire1 through unconventional *HAC1* splicing. Agarose gel electrophoresis results showed that the drug combination markedly reduced *HAC1* splicing efficiency compared with single-drug groups and the control, indicating impaired UPR capacity. Nevertheless, combination treatment did not completely block *HAC1* splicing. This observation is analogous to the findings reported by Halbleib et al. (2017). Upon treatment with 4 mM DTT for 1 h, *HAC1* splicing was substantially impaired but not fully abrogated in the mutant compared with the wild type. Similarly, Wang et al. (2025) characterized *HAC1* splicing in *C. albicans* treated with the Ire1 inhibitor 4μ8C and the ER stress inducer tunicamycin. Treatment with 4μ8C at 5–80 μg/mL partially inhibited tunicamycin-triggered *HAC1* splicing, as the spliced *HAC1*_i_ band remained detectable, whereas complete blockade was only achieved at the higher concentration of 160 μg/mL. 4μ8C potentiates the antifungal potency of the combined regimen against drug-resistant *C. albicans*. Given its specific suppression of Ire1, we further infer that the antifungal activity of this combination is closely associated with compromised function of the Ire1-mediated UPR pathway, which eventually triggers collapse of this pathway. Ire1 has also been reported to sense both protein misfolding and membrane lipid perturbation simultaneously, coordinating ER expansion and membrane remodeling to link protein homeostasis with lipid metabolism networks (Ho et al., 2020). In *C. albicans*, changes in cellular lipid content (such as sterol and sphingolipid profiles) not only affect the structural stability of biofilms but also modulate biofilm-associated antifungal drug resistance, which merits further investigation (Alim et al., 2018; Ottaviano et al., 2025). Besides, under FCZ monotherapy, the expression of *IRE1*, *KAR2*, *SEC61*, and *ERO1* was elevated, which may reflect compensatory UPR activation in response to FCZ-triggered ER stress, aiming to restore ER homeostasis by enhancing protein-folding capacity. In contrast, *HAC1* splicing was suppressed upon BBR-FCZ combination treatment, and the expression of these UPR target genes was markedly reduced, especially under high-dose combination treatment. We speculate that sustained combined drug pressure exceeded the compensatory capacity of the UPR, resulting in the failure of the adaptive stress response and ultimately contributing to the synergistic antifungal effect. This trend is consistent with the inhibitory effect of the Ire1 inhibitor 4μ8C on *KAR2* and *SEC61* expression in *C. albicans* as reported previously (Wang et al., 2025). These data suggest that the Ire1-mediated UPR pathway is potentially involved in the synergistic antifungal response, although direct genetic validation of Ire1 inhibition is still lacking.

Persistent and unresolved ER stress is generally considered to trigger autophagic responses in yeast (Song et al., 2017; Cao et al., 2021; Molinari, 2021). In yeast, autophagosomes form at ER-vacuole contact sites, and complete autophagy relies on the ordered assembly of multiple Atg proteins (Yu et al., 2015; Nakatogawa, 2020). Autophagy activation was observed in *C. albicans* following combination-drug treatment using MDC staining and TEM. In the high-dose combination groups, partial impairment of vacuolar structural integrity was observed, with diffusely distributed C-DCFDA fluorescence signals and a significantly elevated proportion of fluorescence-positive cells. This observation aligns with previous findings that HsAFP1 induces autophagy and impairs vacuolar function in *S. cerevisiae* (Struyfs et al., 2020). Severe defects in vacuolar fusion have also been identified in the *ire1Δ/Δ* strain (Du et al., 2023). Substance leakage resulting from vacuolar fragmentation is considered indispensable for driving yeast cell death, serving as a critical checkpoint for entry into an irreversible lethal process (Kim et al., 2012). Combined treatment also significantly increased the transcript levels of *ATG1*, *ATG8* and *ATG12*, as well as the protein abundance of Atg1. Notably, autophagy exerts dual roles in determining cell fate (Ding et al., 2007). The antimicrobial peptide Scyampcin_44-63_ induces ER stress in *C. albicans*, accompanied by marked vacuolar expansion and the onset of autophagic cell death, as reflected by the ability of 3-MA to considerably delay its fungicidal rate (Zhou et al., 2023). Otilonium bromide has been reported to induce cytotoxic autophagy in *C. albicans*, with RAPA enhancing its antifungal efficacy and the autophagy inhibitor wortmannin partially abrogating this effect (Zhen et al., 2024). In the present study, the autophagy inhibitor 3-MA partially reversed the growth inhibition induced by high-dose combination treatment, suggesting that activated autophagy may shift from a homeostatic adaptive response toward a pro-death phenotype under high drug pressure. Nevertheless, more robust genetic evidence, such as *ATG1* or *ATG8* deletion-rescue experiments, is required to formally establish this causal relationship.

Overall, the synergistic antifungal efficacy of BBR combined with FCZ against clinically resistant *C. albicans* appears to be associated with functional impairment of the Ire1-mediated UPR pathway, though our data do not establish direct Ire1‑mediated causality. Especially, the presented data do not establish that impaired Ire1 signaling directly induces autophagy. A full acknowledgment of these limitations will facilitate further mechanistic validation and translational investigation in future studies. Although the precise causal linkage between UPR dysfunction and autophagy induction remains unclarified, the present exploratory findings provide a preliminary theoretical basis for the development of novel ER stress-targeted antifungal combination strategies.

## Data Availability

The original contributions presented in the study are included in the article/Supplementary material, further inquiries can be directed to the corresponding author.

## Footnotes

1
https://string-db.org/

2
https://pubchem.ncbi.nlm.nih.gov/

3
https://www.uniprot.org/uniprotkb

4
https://www.rcsb.org/
