# Supplementary material for: Berberine-fluconazole synergy against drug-resistant Candida albicans: association with disruption of the Ire1-mediated UPR pathway
Source: Front Microbiol. 2026 Oct 9;17:1965937. doi: 10.3389/fmicb.2026.1965937 (PMC13690794; doi:10.3389/fmicb.2026.1965937)
Supplement: Supplementary file 1 [file Table_1.DOCX]

**Table S1** Primers for RT-qPCR

| Primers | Sequence (5' to 3') | Tm (℃) | Product Size (bp) |
| --- | --- | --- | --- |
| *ACT1*-F | TCTGTCGGCAGTGGTTTCAA | 56.80 | 120 |
| *ACT1*-R | GCCTTGCACAAGTACACGTAG | 56.40 |  |
| *IRE1*-F | GCAGATTGCCCAGTTGTTGG | 60.04 | 136 |
| *IRE1*-R | ACGAAAAACAATGGCGGAGC | 60.04 |  |
| *KAR2*-F | TTCACCGAAGCACGCATTTG | 60.04 | 231 |
| *KAR2*-R | GCTTGATTCTTGGCAGCGTC | 60.18 |  |
| *SEC61*-F | CAACAGCGTGTGATGTGGAC | 59.76 | 182 |
| *SEC61*-R | ATCCCCGAGGAGACGATAGG | 59.96 |  |
| *ERO1*-F | ATGATGCCGACAATGGTGGT | 60.03 | 221 |
| *ERO1*-R | AGCTTGGGACACTAAGGCAT | 59.00 |  |
| *ATG1*-F | GGTGATGCCCAAAATACCAATAATG | 59.01 | 192 |
| *ATG1*-R | ATCGATACATTTATAAACCGTGGCA | 59.01 |  |
| *ATG2*-F | AGTAGCATTCCTGTTGGCCC | 60.03 | 171 |
| *ATG2*-R | CTTGTTCGCCTTTTCGGTGG | 60.04 |  |
| *ATG8*-F | GCCAGAATTGCTCAGAGGTT | 58.17 | 220 |
| *ATG8*-R | TGATTAATGCAGCGGTTGGG | 58.90 |  |
| *ATG12*-F | GTTCAACACCGTCGATTCAA | 56.69 | 155 |
| *ATG12*-R | TATCTGCTCGTCAGGCAATG | 57.77 |  |

**Table S2** Antibodies for Western blotting

| Antibody Name | Antibody Type | Source | Dilution |
| --- | --- | --- | --- |
| Atg1 | Rabbit polyclonal antibody | Daian, Wuhan, China | 1:500 |
| β-Tubulin | Mouse monoclonal antibody | Beyotime, Shanghai, China | 1:500 |
| HRP-conjugated goat anti-rabbit IgG | Goat monoclonal antibody | Proteintech, Wuhan, China | 1:10,000 |
| HRP-conjugated goat anti-mouse IgG | Goat monoclonal antibody | Proteintech, Wuhan, China | 1:10,000 |

Note: PVDF membranes were incubated with primary antibodies overnight at 4 °C, followed by incubation with secondary antibodies at 25 °C for 1 h.
